# Continuous Glucose Monitoring in Primary Care: Multidisciplinary Pilot Implementation Study

**DOI:** 10.2196/69061

**Published:** 2025-06-18

**Authors:** Alyssa H Zadel, Katia Chiampas, Katrina Maktaz, John G Keller, Kathy W O'Gara, Leonardo Vargas, Angela Tzortzakis, Micah J Eimer, Emily D Szmuilowicz

**Affiliations:** 1 Division of Cardiology, Northwestern Medicine, Chicago, IL, United States; 2 Medical College of Wisconsin, Milwaukee, WI, United States; 3 Johns Hopkins University, Baltimore, MD, United States; 4 Nova Southeastern University, Fort Lauderdale, FL, United States; 5 Department of Pharmacy, Northwestern Medicine, Chicago, IL, United States; 6 Division of Endocrinology, Metabolism, and Molecular Medicine, Northwestern Medicine, Chicago, IL, United States; 7 Division of Internal Medicine, Northwestern Medicine, Chicago, IL, United States; 8 Northwestern Medicine, Chicago, IL, United States; 9 Division of Endocrinology, Metabolism and Molecular Medicine, Northwestern University Feinberg School of Medicine, 645 N Michigan Ave, 530-24, Chicago, IL, 60611, United States, 1 312 695 7970, 1 312 926 8693

**Keywords:** continuous glucose monitoring, diabetes education, primary care physicians, multidisciplinary team, type 2 diabetes

## Abstract

**Background:**

Continuous glucose monitoring (CGM) is used to assess glycemic trends and guide therapeutic changes for people with diabetes. We aimed to increase patient access to this tool by equipping primary care physicians (PCPs) to accurately interpret and integrate CGM into their practice via a multidisciplinary team approach.

**Objective:**

The primary objective of this study was to evaluate the feasibility and effectiveness of integrating CGM into primary care clinics using a multidisciplinary approach that included a clinical pharmacist (PharmD) and a certified diabetes care and education specialist (CDCES).

**Methods:**

Eighteen PCPs received a 1-hour video training module from an endocrinologist teaching a systematic stepwise approach to CGM interpretation. Patient inclusion criteria included type 2 diabetes mellitus, ≥18 years old, hemoglobin A_1c_ (HbA_1c_) ≥8% or concern for hypoglycemia, and no previous CGM use or an endocrinology visit in the past year. Patients saw physician extenders (CDCES or a PharmD) for professional CGM placement and education on nutrition, medication administration, and physical activity goals based on the PCP’s recommendations. The CDCES or PharmD reviewed CGM data with patients and collaborated with PCPs to adjust the care plan, informed by the systematic stepwise approach to CGM interpretation. Patients either converted to personal CGM if desired or had a second professional CGM device placed after ≥1 month from the initial professional CGM placement and obtained a postintervention HbA_1c_ measurement at ≥3 months from the initial HbA_1c_ measurement. The primary outcomes were time in range, HbA_1c_, and average time from referral to the first CGM device placement. Follow-up continued with the CDCES or PharmD until patients met the study discharge criteria of HbA_1c_ level ≤7%. Paired *t* tests with 1-sided *P* values were used to assess changes in glucose metrics from the initial to postintervention measurements. The McNemar test was used to determine the significance of change in patients meeting the goal of ≥70% time in the target range of 70-180 mg/dL.

**Results:**

The CGM users (n=46) had a mean (SD) age of 62.39 (14.57) years, and 14/46 participants (30%) were female. The mean (SD) time in range increased by 28.06%, from 43.25% (33.41%) at baseline to 71.31% (25.49%) postintervention (*P*<.001), due to reduced hyperglycemia. The proportion of CGM users meeting the consensus target of the time in range ≥70% increased from 23.81% to 57.14% (*P*<.001). Postintervention HbA_1c_ decreased by an average of 2.37%, from 9.68% (1.78%) to 7.31% (1.32%; *P*<.001).

**Conclusions:**

The integration of CGM into primary care clinics to increase patient access is feasible and effective using a multidisciplinary approach.

## Introduction

In the United States, 38.4 million people (11.6% of the US population) are living with diabetes [1]. As the eighth leading cause of death in the US [2], diabetes continues to be a significant health concern, with increased needs for large-scale, comprehensive strategies for diagnosis and medical management [3]. The 2025 American Diabetes Association Standards of Care in Diabetes [4] recommend quarterly hemoglobin A_1c_ (HbA_1c_) testing, in addition to blood glucose monitoring and continuous glucose monitoring (CGM) for people who are treated with any type of insulin therapy. CGM is a technology that has transformed modern diabetes care, elucidating daily glycemic trends in a way that neither HbA_1c_ nor blood glucose monitoring testing can, by characterizing glycemic profiles in real time over the course of the day and glycemic trends over periods of time [5]. The use of CGM increases the time spent in the target range of 70‐180 mg/dL (time in range) while reducing HbA_1c_ [6]. This is achieved through coupling the proper interpretation of CGM results with medication and lifestyle adjustments recommended by health care providers [7]. As CGM is traditionally used by endocrinologists [8], the integration of CGM into patient care is often slowed by the shortage of endocrinologists [9]. In addition, many individuals living with diabetes are cared for by primary care physicians (PCPs) rather than endocrinologists [10]. It is thus of paramount importance to develop approaches that enable the integration of CGM into primary care, so that access to CGM is equitably afforded to all people living with diabetes and not just those under the care of an endocrinologist.

Integrating CGM into primary care holds a promising future but has some nuances that must be addressed to be successful [11]. First, patients must have access to CGM technology. Implementing a care team that is trained to interpret CGM is pivotal to any workflow that incorporates CGM into primary care and reduces therapeutic inertia [12]. Additionally, the care team, including PCPs, must be formally educated on how to interpret and use CGM data to optimize clinical care [13]. Although any PCP can prescribe CGM, they must understand how to interpret results and adjust the care plan based on the data provided by CGM, in order for the use of CGM to be clinically effective. In this pilot program, we addressed these concerns by implementing a systematic workflow with a multidisciplinary team including PCPs, a certified diabetes care and education specialist (CDCES), and clinical pharmacist (PharmD) who were all taught a systematic and stepwise approach to CGM interpretation by an endocrinologist. The CDCES and PharmD regularly communicated with the PCPs regarding clinical management. The goal of this study is to demonstrate feasibility of a systematic workflow that enables the integration of CGM into primary care while alleviating the burden on PCPs, and as a result improve diabetes management strategies in a primary care setting.

## Methods

### Ethical Considerations

Per our institutional policy, this met the definition of quality improvement and not human subjects research, and therefore, did not require institutional review board review or oversight. Participants had the ability to opt out and were not compensated. Data were deidentified.

### Participants

Eighteen PCPs as well as the participating CDCES and PharmD from two academic-affiliated community-based outpatient primary care clinics within multispecialty urban practices were offered a 1-hour recorded training module led by an endocrinologist regarding a systematic and stepwise approach to CGM interpretation, based on published methodology [14], followed by a live question and answer session with an endocrinologist. The CDCES was a registered nurse, and the PharmD operated under a collaborative practice agreement. PCPs who successfully completed training on CGM interpretation could refer eligible patients to the CDCES or PharmD, who then collaborated with the PCP to modify the diabetes treatment regimen. Patient inclusion criteria included type 2 diabetes mellitus, age ≥18 years, HbA_1c_ ≥8%, or concern for hypoglycemia (Table 1). The exclusion criteria included the past use of CGM or an endocrinology visit in the past year. Three patients were included with HbA_1c_ <8% due to concern for hypoglycemia.

**Table 1.** Patient demographics.

Category	Patients, n (%)
Age group, years	
30‐39	4 (9)
40‐49	7 (15)
50‐59	10 (22)
60‐69	9 (19)
>70	16 (35)
Sex	
Male	32 (70)
Female	14 (30)
Duration of disease	
<1 year	6 (13)
1‐5 years	13 (28)
6‐10 years	17 (37)
>10 years	10 (22)

### Systematic Workflow

**Figure 1.**
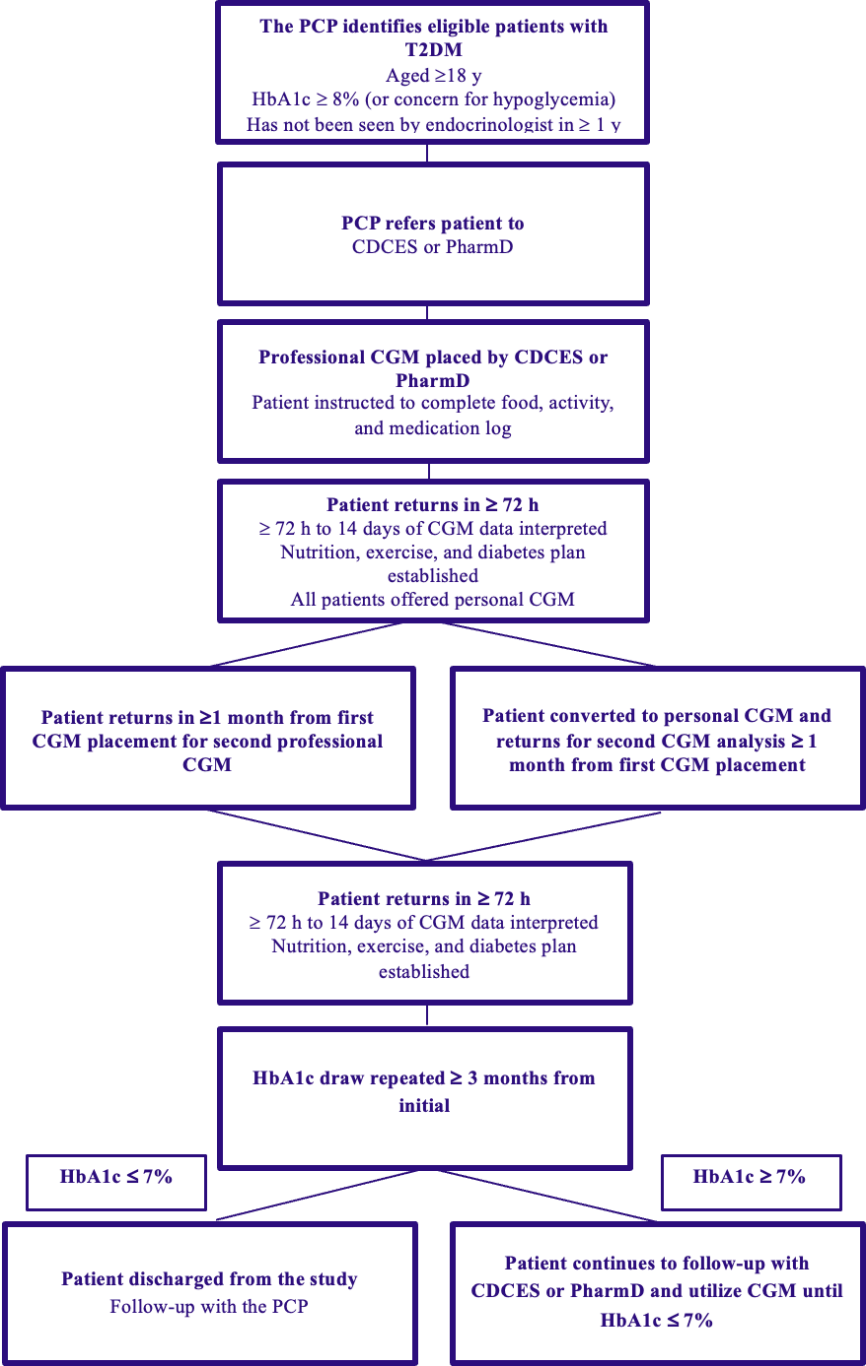
Systematic workflow for CGM implementation in a primary care practice. CDCES: certified diabetes care and education specialist; CGM: continuous glucose monitoring; HbA_1c_: hemoglobin A_1c_; PCP: primary care physician; PharmD: clinical pharmacist; T2DM: type 2 diabetes mellitus.

Before referral, the PCP provided basic CGM education to the patient and explained the clinical benefits of wearing a professional CGM device (Figure 1). The PCP explained the roles of the CDCES and PharmD to provide further CGM education and manage follow-up in collaboration with the PCP. The CDCES or PharmD met with the patient for 30‐60 minutes to provide basic diabetes education on nutrition and lifestyle, discuss potential options for medication adjustment based on the PCP’s recommendations, and the plan to monitor glycemic trends with CGM. A tailored plan was created to help patients effectively manage their diabetes through mutually agreed upon self-care behaviors such as healthy coping, healthy eating, physical activity, medication monitoring, problem solving, and risk reduction. Food logs were reviewed with patients to allow them to understand how food choices impact their glucose levels. The CDCES provided education on balanced and carbohydrate-consistent meals. Education on concepts were discussed, including decreased portion size, increasing nonstarchy vegetables, moderate carbohydrate portions, modifications of meal order (eating protein before carbohydrate when possible), and increasing intake of nutrient-dense food options. Patient-specific titration of medications including insulin, GLP-1 receptor agonists, and oral medications were completed based on the CGM data review. The CDCES and PharmD ensured patient understanding of glucose target ranges and indications to notify the team if glucose was above or below the target. The PharmD or CDCES provided injection training when necessary. Patients were educated on signs, symptoms, and treatment of hypoglycemic episodes. A professional CGM device was placed at the first or second visit with either the PharmD or CDCES. The CGM device used in this pilot was the Dexcom G6 Pro (San Diego, CA), with results blinded or unblinded to the patient depending on patient preference. All patients who participated in the study used the unblinded mode to view their glycemic data in real time, allowing adjustment and reinforcement in real time of the recommended lifestyle modifications. In addition to the CGM placement, the patient was asked to complete a food, activity, and medication log and to return it in ≥72 hours. At the return visit, the CGM device was removed, data were uploaded, and the patient was given a copy of the ambulatory glucose profile report. The following CGM parameters were recorded: time in range, time above range, time below range, mean glucose, glucose management indicator, and coefficient of variation. The CGM report was sent to the PCP, and therapeutic modifications were generated by the PCP in collaboration with the physician extender. The CDCES or PharmD discussed adjustments to the personalized care plan with the patient. Appropriate billing codes (Table 2) were entered by the CDCES or PharmD and PCP. If patients desired to do so, they were converted to personal CGM. If not, a second professional CGM device was placed by the CDCES or PharmD no sooner than 1 month after the initial professional CGM device was placed. A postintervention HbA_1c_ measurement was obtained ≥3 months after the initial HbA_1c_ measurement. Patients continued to meet with the CDCES or PharmD in between PCP appointments. Follow-up continued with the CDCES or PharmD until patients met the study discharge criteria of HbA_1c_ ≤ 7%.

**Table 2.** Billing codes.

Code	Entered by	Purpose
95250	CDCES^a^ or PharmD^b^	Placement, removal, upload of data
95251	PCP^c^	CGM^d^ interpretation
95249	CDCES or PharmD	Training on personal CGM
G0108	CDCES	Diabetes education
99211	PharmD	Pharmacist visit

a CDCES: certified diabetes care and education specialist.

b PharmD: clinical pharmacist.

c PCP: primary care physician.

d CGM: continuous glucose monitoring.

### Statistical Analysis

The primary outcomes were time in range, HbA_1c_, and average time from referral to first CGM device placement. Descriptive statistics including the mean (SD) and percentages were used to analyze the age and sex of participants, respectively. Paired 1-tailed *t* tests with 1-sided *P* values were used with 2-sided 95% CIs to determine the differences in initial and postintervention values for mean glucose, glucose management indicator, coefficient of variation, HbA_1c_, time in range, time above range, and time below range. The McNemar test was used to determine the significance of change in patients meeting the goal of ≥70% time in the target range of 70‐180 mg/dL, according to the International Consensus on Time in Range [15], from the initial compared to postintervention CGM use. A Kaplan–Meier curve was generated to analyze the time to discharge and determine the median number of days participants remaining in the study to meet discharge criteria. Analyses were conducted using JASP 0.18.3 (JASP Team; version 0183, Intel) and the criterion for statistical significance was *P*<.05.

## Results

### Participant Statistics

The mean (SD) age of CGM users (n=46) was 62.39 (14.57) years, and 14 out of 46 participants (30%) were female. A total of 3 individuals were lost to follow-up. The time in range increased significantly by 28.06%, from 43.25% (33.41%) at baseline to 71.31% (25.49%) postintervention (*P*<.001), due to reduced hyperglycemia (Table 3). There was a significant 42.14 mg/dL decrease in the average mean glucose, from 201.71 (51.98) mg/dL to 159.57 (30.68) mg/dL. The percentage of patients meeting the goal time in range (70‐180 mg/dL) ≥70% [15] increased significantly from 23.81% to 57.14% (*P*<.001). There was no significant change in the time below range (mean 0.12%, SD 0.33% vs mean 0.43%, SD 0.94%; *P*=.10). Postintervention, HbA_1c_ decreased by an average (SD) of 2.37% from 9.68% (1.78%) to 7.31% (1.32%; *P*<.001). Similarly, the glucose management indicator decreased an average (SD) of 0.90% from 8.00% (1.18%) to 7.10% (0.74%).

**Table 3.** Changes in standardized continuous glucose monitoring metrics.

Parameters	Baseline mean (SD)	Follow-up mean (SD)	Postintervention change, mean (95% CI)	*P* value
Mean glucose (mg/dL)	201.71 (51.98)	159.57 (30.68)	−42.14 (−56.92 to −27.36)	<.001
Glucose management indicator (%)	8.00 (1.18)	7.10 (0.74)	−0.90 (−1.20 to −0.59)	<.001
Coefficient of variation (%)	21.15 (6.79)	21.65 (7.38)	0.50 (−0.79 to 1.79)	.77
Hemoglobin A_1c_ (%)	9.68 (1.78)	7.31 (1.32)	−2.37 (−2.88 to −1.86)	<.001
Percentage time in range (70‐180 mg/dL)	43.25 (33.41)	71.31 (25.49)	28.06 (18.48 to 37.64)	<.001
Percentage time above range	56.90 (33.74)	28.57 (26.27)	−28.33 (−37.90 to −18.75)	<.001
Percentage time below range	0.12 (0.33)	0.43 (0.94)	0.31 (0.94 to 0.53)	.10

### Systematic Workflow Outcomes

**Figure 2.**
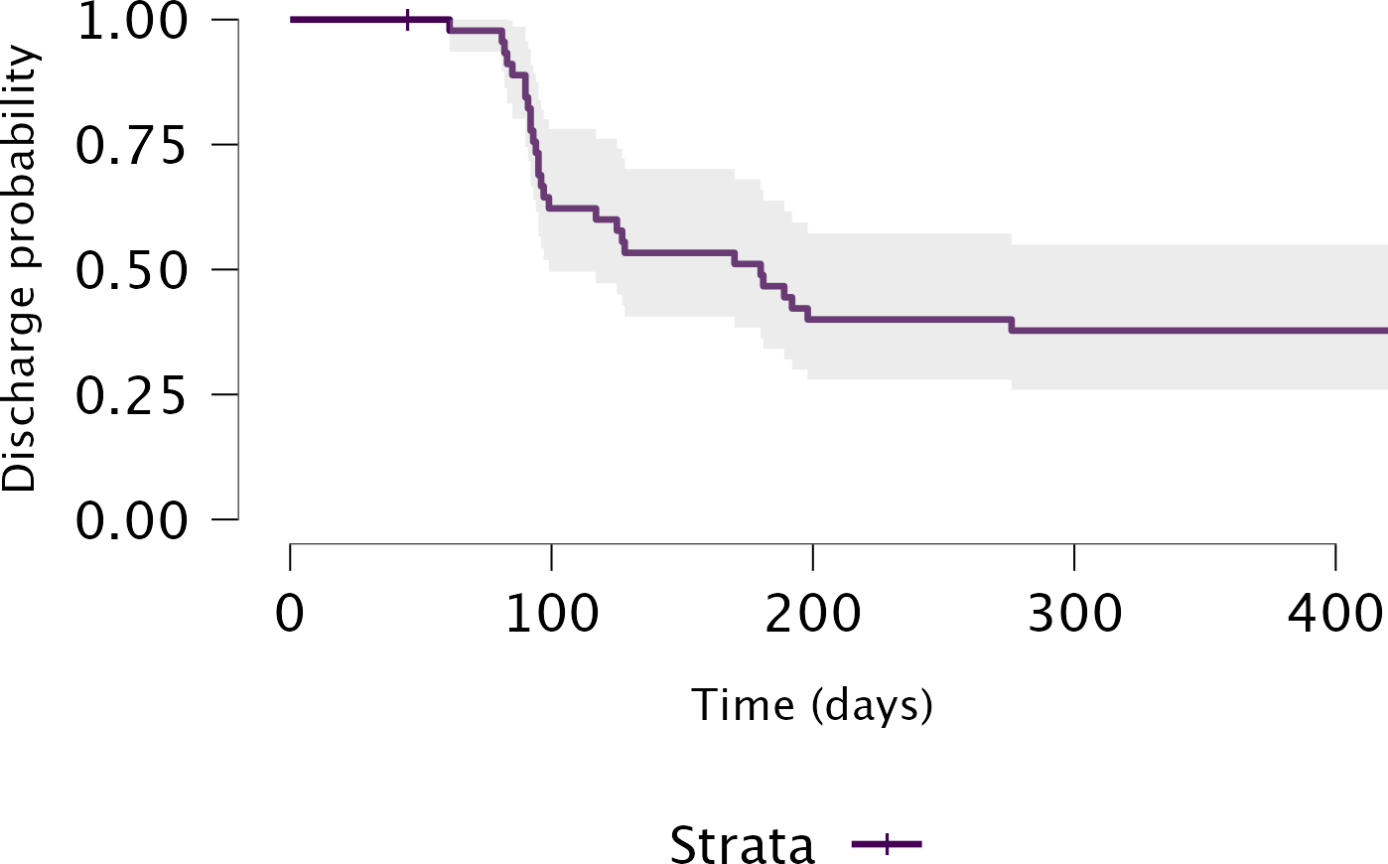
Kaplan-Meier curve for discharge probability.

The average time from referral to the first CGM device placement was 21.10 (IQR 6‐24) days. The average time between the first and second CGM device placement or, if desired, conversion to personal CGM was 66.79 (IQR 43.50‐78.75) days. Out of 46 people, 21 (46%) people converted to personal CGM devices. Out of 46 people, 28 (61%) patients met the goal of HbA_1c_ ≤7% to be discharged from the pilot program. The median number of days a patient remained in the study from referral to discharge was 180 days (Figure 2).

## Discussion

### Principal Results

In this pilot study, we demonstrated the feasibility of implementation of a reproducible systematic workflow for the incorporation of CGM into primary care, guided by teaching regarding the systematic interpretation of CGM from endocrinology. Our multidisciplinary approach reduced the burden of implementing CGM into primary care, particularly reducing the workflow of the PCP by using physician extenders to oversee care. Consistent with other studies [7,16], the use of CGM significantly improved glycemic metrics in this pilot program, including an improved time in range and decreased HbA_1c_ without an associated increase in hypoglycemia. The study showed that access to CGM via our systematic workflow occurred in less than 1 month, compared to an average wait time of 227 days [17] to be seen by endocrinology, suggesting that this type of systematic workflow could decrease time to uptake of diabetes technology. Our findings are strengthened by the fact that this systematic workflow, informed by a published stepwise approach to CGM interpretation, could be easily reproduced at other centers. The generalizability of our findings is limited by the fact that our study was a small pilot study limited to two clinical sites. Additionally, the systematic workflow used in the study is limited by the fact that not all primary care clinics have access to physician extenders such as a CDCES or a PharmD.

### Future Directions

Future directions include the expansion of the systematic workflow to additional sites with the goal of expanding generalizability of our findings. Future studies could evaluate whether the conversion of patients to personal CGM will facilitate the long-term use of CGM technology.

### Conclusions

CGM is a powerful tool in modern diabetes management, yet access to endocrinologists with specialized training in use of this technology is curtailed by the limited supply of endocrinologists [18]. PCPs, who see most patients with type 2 diabetes [19], are similarly oversaturated and may lack expertise in CGM interpretation [20]. However, the use of CGM in primary care is increasing as the shortage of endocrinologists continues to present challenges [18,20]. In order to optimize the clinical utility of CGM technology, the prescribing physician must be able to efficiently and accurately interpret CGM data. While educational materials regarding CGM use and CGM data interpretation targeted toward PCPs are expanding [21], this pilot program allowed PCPs to have a structured training experience with an endocrinologist with the goal of increasing familiarity and comfort with the integration of this technology into a primary care practice. Additionally, the program alleviated the burden on PCPs through multidisciplinary collaboration with physician extenders. This collaborative approach not only encourages more efficient diabetes management but also empowers patients with a better understanding of their personalized glycemic trends and the impact of therapeutic adjustments.

## Abbreviations

CDCES: certified diabetes care and education specialist
CGM: continuous glucose monitoring
HbA_1c_: hemoglobin A_1c_
PCP: primary care physician
PharmD: clinical pharmacist
